# Port type is a possible risk factor for implantable venous access port-related bloodstream infections and no sign of local infection predicts the growth of gram-negative bacilli

**DOI:** 10.1186/s12957-015-0707-2

**Published:** 2015-09-30

**Authors:** Jui-Feng Hsu, Hsu-Liang Chang, Ming-Ju Tsai, Ying-Ming Tsai, Yen-Lung Lee, Pei-Huan Chen, Wen-Chieh Fan, Yu-Chung Su, Chih-Jen Yang

**Affiliations:** Department of Internal Medicine, Kaohsiung Municipal Ta-Tung Hospital, Kaohsiung Medical University, No. 68 Chunghwa 3rd Road, Cianjin District, 80145 Kaohsiung City, Taiwan; Department of Surgery, Kaohsiung Municipal Ta-Tung Hospital, Kaohsiung Medical University Hospital, Kaohsiung Medical University, Kaohsiung, Taiwan; Division of Pulmonary and Critical Care Medicine, Department of Internal Medicine, Kaohsiung Medical University Hospital, Kaohsiung, Taiwan; School of Medicine, College of Medicine, Kaohsiung Medical University, Kaohsiung, Taiwan

**Keywords:** Venous port, Infection

## Abstract

**Background:**

Implantable venous access port (IVAP)-related blood stream infections (BSIs) are one of the most common complications of implantable venous ports. The risk factors and pathogens for IVAP-related BSIs are still controversial.

**Methods:**

We retrospectively reviewed all patients who received IVAPs at a Hospital in Taiwan from January 1, 2011 to June 31, 2014. Two types of venous port, BardPort® 6.6 fr (Bard port) and Autosuture Chemosite® 7.5 fr (TYCO port) were used. All patients with clinically proven venous port-related BSIs were enrolled.

**Results:**

A total of 552 patients were enrolled. There were 34 episodes of IVAP-related BSIs during the study period for a total incidence of 0.177 events/1000 catheter days. Port type (TYCO vs. Bard, HR = 7.105 (95 % confidence interval (CI), 1.688–29.904), *p* = 0.0075), age > 65 years (HR = 2.320 (95 % CI, 1.179–4.564), *p* = 0.0148), and lung cancer (HR = 5.807 (95 % CI, 2.946–11.447), p < 0.001) were risk factors for port infections. We also found that no local sign of infection was significantly associated with the growth of gram-negative bacilli (*p* = 0.031).

**Conclusions:**

TYCO venous ports, age > 65 years, and lung cancer were all significant risk factors for IVAP-related BSIs, and no sign of infection was significantly associated with the growth of gram-negative bacilli.

## Background

Venous port implantation is widely used for the safe delivery of systemic chemotherapy in patients with cancer. However, various complications have been reported with an overall complication rate ranging from 0.4 to 29 % [1–4]. The major complications of implantable venous access port (IVAP) placement include infection, thrombosis, catheter obstruction, extravasation, and catheter migration [1, 5, 6], of which IVAP-related infection is the most common. Catheter-related blood stream infections (BSIs) have been reported in 2.4 to 16.4 % of cases [4, 7–11]. Several studies have reported factors that increase infectious port complications, with one of the most significant being hematologic malignancy. However, to the best of our knowledge, only a few studies have systematically analyzed the risk factors for IVAP-related blood infections. Samaras et al. reported that port-associated infections are mostly observed in younger patients with hematologic neoplasms [12]. Recently, Shim et al. concluded that hematologic malignancy and receiving palliative chemotherapy were independent risk factors for IVAP-related BSIs [8]. In addition, a case control study performed by Lee et al. showed that prolonged catheter placement was a risk factor for catheter-related BSIs and that the risk was lower in patients with primary gastrointestinal cancer than in other types of cancer [10]. However, to date, no studies have investigated whether the type of venous port is a possible risk factor for venous port infections. The aim of this retrospective study, therefore, was to investigate the risk factors for IVAP-related BSIs in a university affiliated hospital in Taiwan. We also reviewed the related literature.

## Methods

The study population included all patients who received IVAPs for chemotherapy at Kaohsiung Municipal Ta-Tung Hospital from January 1, 2011 to June 30, 2014. The IVAPs included the BardPort® 6.6 fr implantable port (Bard, NJ, USA (Bard port)) and the Autosuture Chemosite® Fr.7.5 port (Tyco Healthcare Group, CT, USA (TYCO port)). The Bard port was made of silicon, and the TYCO port was made of polyurethane. All IVAPs were placed by surgeons under local anesthesia and aseptic conditions without the use of prophylactic antibiotics. The vessel cutdown method was used for catheter cannulation. After venostomy, the distal end of the entry vessel was controlled and the catheter was inserted via the superior vena cava. The cephalic vein was the first choice for entry, and the subclavian vein was used as the point of the entry if the cephalic vein was difficult to approach. All locations of the implanted venous ports were confirmed by fluoroscopy and postoperative X-rays. The surgeon ensured that all of the tips of the venous catheters were located at the junction of the superior vena cava and right atrium (cavo-atrial junction) intraoperatively. IVAPs placed in the femoral veins were excluded.

For all patients with signs and symptoms of IVAP-related BSIs, we routinely perform aerobic and anaerobic microorganism cultures with whole blood samples. Blood culture results that were positive for a fungus or bacteria were recorded. The possibility of a contaminated blood culture was determined after agreement by two physicians (Dr. Hsu JF and Dr. Chang HL). The catheter tips of the removed IVAPs were placed on agar plates and sent to our laboratory for microbiology studies.

An IVAP-related BSI was defined according to the definition reported by Liaw et al. [13] as (1) clinical features of infection, fever, and chills but no identifiable focus of infection elsewhere and (2) isolation of the same organism from the catheter tip and peripheral blood cultures. A probable IVAP-related BSI was considered on the basis of fever and chills following port flush and a positive blood culture result but no identifiable focus of infection elsewhere. One of the following criteria was also required: (1) isolation of the same organism from the port and peripheral blood cultures at the same time but a negative catheter tip culture and (2) isolation of the same organism from peripheral blood cultures when fever and chills occurred following port flush but a negative catheter tip culture. Local infection was defined as the presence of signs of local inflammation, including erythema, warmth, tenderness, and pus formation. When signs of a local infection were noted, wound swab cultures were obtained. To calculate the incidence rate (events per 1000 catheter days), the duration of IVAP catheter use was calculated using the last day of the patients’ medical records instead of the day of IVAP removal.

The Institutional Review Board (IRB) of Kaohsiung Medical University Hospital (KMUH) approved this study (KMUH-IRB-20140366) according to Taiwan national regulations. Considering the retrospective nature of the study, we could not obtain patient consent for the use of clinical data, and the IRB of KMUH waived the need for written informed consent from the participants. In addition, information in the patient records was anonymized and de-identified prior to analysis.

Data were entered and analyzed using JMP statistical software (version 9.0, SAS Institute Inc., Cary, NC, USA). Demographic data, underlying cancer, and related covariates were compared between the groups with and without port infections using Fisher’s exact test for categorical variables and the Wilcoxon rank sum test for continuous variables. To identify the major factors associated with port infections, Cox multivariate logistic regression analysis with stepwise variable selection was performed. Statistical significance was set at *p* < 0.05.

## Results

**Fig. 1:**
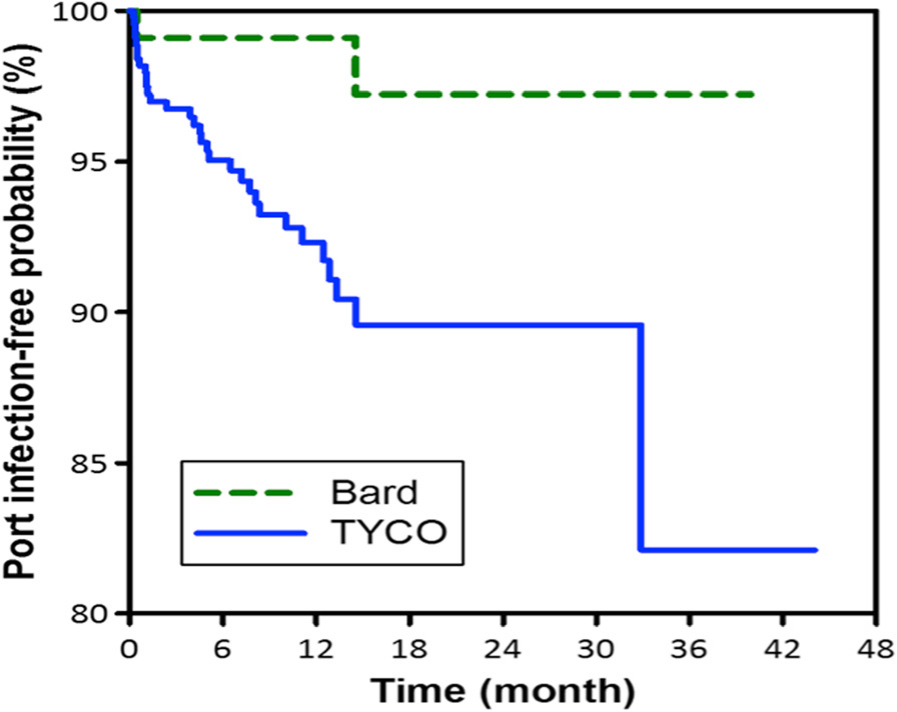
The TYCO venous port had a higher infection rate than the Bard type (*p* = 0.0185)

A total of 552 patients were enrolled. There were 34 episodes of IVAP-related BSIs during the study period, for a total incidence of IVAP-related BSIs of 0.177 events/1000 catheter days. Table 1 shows the clinical characteristics of the patients according to the port used, and the TYCO venous port had a higher infection rate than the Bard type (*p* = 0.0185) (Fig. 1).

**Table 1:** Clinical characteristics of the patients using different ports

Variables	All patients	Bard	TYCO	*P* value
*N*	552	113 (20 %)	439 (80 %)	
Sex—*n* (%)				0.0003
Female	317 (57 %)	48 (42 %)	269 (61 %)	
Male	235 (43 %)	65 (58 %)	170 (39 %)	
Age—mean ± SD	59.9 ± 12.3	63.6 ± 11	58.9 ± 12.4	0.0002
Age—*n* (%)				0.0050
Age ≤ 65	369 (67 %)	63 (56 %)	306 (70 %)	
Age > 65	183 (33 %)	50 (44 %)	133 (30 %)	
Malignancy (indication for the port)—*n* (%)				<0.0001
Lung cancer	105 (19 %)	34 (30 %)	71 (16 %)	
Head and neck tumor	29 (5 %)	4 (4 %)	25 (6 %)	
Breast cancer	132 (24 %)	8 (7 %)	124 (28 %)	
Esophageal cancer	5 (1 %)	0 (0 %)	5 (1 %)	
Gastric cancer	22 (4 %)	7 (6 %)	15 (3 %)	
Colorectal cancer	114 (21 %)	28 (25 %)	86 (20 %)	
Urological cancer	55 (10 %)	14 (12 %)	41 (9 %)	
Ovary cancer and cervical cancer	47 (9 %)	9 (8 %)	38 (9 %)	
Hepatobiliary and pancreatic tumor	9 (2 %)	4 (4 %)	5 (1 %)	
Leukemia and lymphoma	25 (5 %)	3 (3 %)	22 (5 %)	
Other malignancies	9 (2 %)	2 (2 %)	7 (2 %)	
Malignancy (lung cancer or others)—*n* (%)				0.0008
Lung cancer	105 (19 %)	34 (30 %)	71 (16 %)	
Other malignancies	447 (81 %)	79 (70 %)	368 (84 %)	
Surgeon—*n* (%)				0.0031
Surgeon A	142 (26 %)	37 (33 %)	105 (24 %)	
Surgeon B	358 (65 %)	74 (65 %)	284 (65 %)	
Other surgeons	52 (9 %)	2 (2 %)	50 (11 %)	
Port infection (+)—*n* (%)	34 (6 %)	2 (2 %)	32 (7 %)	0.0295
Gram-positive bacteria	15 (44 %)	2 (100 %)	13 (41 %)	0.1009
*S. aureus*	13 (38 %)	2 (100 %)	11 (34 %)	0.0639
Gram-negative bacteria	19 (56 %)	1 (50 %)	18 (56 %)	0.8629
*K. pneumoniae*	10 (29 %)	0 (0 %)	10 (31 %)	0.3467
*Candida spp*.	4 (12 %)	0 (0 %)	4 (13 %)	0.5945

Venous port infections were more common in males, those age > 65 years and those with lung cancer (Table 2). In multivariate Cox regression analysis, port type (TYCO vs. Bard, hazard ratio (HR) = 7.105 (95 % confidence interval (CI), 1.688–29.904), *p* = 0.0075), age > 65 years (HR = 2.320 (95 % CI: 1.179–4.564), *p* = 0.0148), and lung cancer (HR = 5.807 (95 % CI, 2.946–11.447), *p* < 0.001) were risk factors for port infections (Table 3).

**Table 2:** Clinical characteristics of the patients with or without port infections

Variables	All patients	Port infection (−)	Port infection (+)	*P* value
*N*	552	518 (94 %)	34 (6 %)	
Gender—*n* (%)				0.0071
Female	317 (57 %)	305 (59 %)	12 (35 %)	
Male	235 (43 %)	213 (41 %)	22 (65 %)	
Age—mean ± SD	59.9 ± 12.3	59.5 ± 12.3	64.9 ± 11.1	0.0132
Age—*n* (%)				0.0312
Age ≤ 65 years	369 (67 %)	352 (68 %)	17 (50 %)	
Age > 65 years	183 (33 %)	166 (32 %)	17 (50 %)	
Malignancy (indication for the port)—*n* (%)				0.0005
Lung cancer	105 (19 %)	88 (17 %)	17 (50 %)	
Head and neck tumor	29 (5 %)	27 (5 %)	2 (6 %)	
Breast cancer	132 (24 %)	130 (25 %)	2 (6 %)	
Esophageal cancer	5 (1 %)	5 (1 %)	0 (0 %)	
Gastric cancer	22 (4 %)	22 (4 %)	0 (0 %)	
Colorectal cancer	114 (21 %)	111 (21 %)	3 (9 %)	
Urological cancer	55 (10 %)	50 (10 %)	5 (15 %)	
Ovary cancer and cervical cancer	47 (9 %)	43 (8 %)	4 (12 %)	
Hepatobiliary and pancreatic tumor	9 (2 %)	9 (2 %)	0 (0 %)	
Leukemia and lymphoma	25 (5 %)	25 (5 %)	0 (0 %)	
Other malignancies	9 (2 %)	8 (2 %)	1 (3 %)	
Malignancy (lung cancer or others)—*n* (%)				<0.0001
Lung cancer	447 (81 %)	430 (83 %)	17 (50 %)	
Other malignancies	105 (19 %)	88 (17 %)	17 (50 %)	
Surgeon—*n* (%)				0.5273
Surgeon A	142 (26 %)	131 (25 %)	11 (32 %)	
Surgeon B	358 (65 %)	339 (65 %)	19 (56 %)	
Other surgeons	52 (9 %)	48 (9 %)	4 (12 %)

**Table 3:** Catheter-related bacteremia-related pathogens in this study

Bacteria	Number
Gram-positive bacteria	15
*Staphylococcus aureus*	13
Oxacillin-susceptible (OSSA)	8
Oxacillin-resistant (ORSA)	5
*Bacillus spp*.	1
*Enterococcus faecium*	1
Gram-negative bacteria	19
*Klebsiella pneumoniae*	10
*Acinetobacter baumannii*	2
*Escherichia coli*	2
*Enterobacter aerogenes*	1
*Pseudomonas aeruginosa*	2
*Salmonella*, group B	1
*Bacteroides ureolyticus*	1
*Candida spp*.	4

We further analyzed the pathogens and found that 19 (55.8 %) cases involved gram-negative bacteria, with *Klebsiella pneumoniae* being the most common pathogen followed by *Acinetobacter baumannii*, *Escherichia coli*, and *Pseudomonas aeruginosa* (Table 4)*.* Fifteen (39.4 %) infections involved gram-positive bacteria, with *Staphylococcus aureus* being the most common pathogen. Oxacillin-sensitive *Staphylococcu*s *aureus* was more common than oxacillin-resistant *Staphylococcus aureus*. In addition, there were four (10.5 %) fungal infections, all of which involved *Candida s*pp. No local infections significantly grew gram-negative bacilli (*p* = 0.031) (Table 5).

**Table 4:** Cox proportional hazard regression analysis to identify the factors associated with port-infection-free survival

Clinical features	Univariate analysis		Multivariate analysis—maximal model		Multivariate analysis—reduced model	
	HR (95 % CI)	*p* value	HR (95 % CI)	*p* value	HR (95 % CI)	*p* value
Port type (TYCO vs. Bard)	4.759 (1.137–19.927)	0.0327	7.643 (1.795–32.535)	0.0059	7.105 (1.688–29.904)	0.0075
Gender (male vs. female)	2.849 (1.408–5.766)	0.0036	1.974 (0.934–4.172)	0.0748		
Age (>65 vs. ≤65 years)	2.262 (1.154–4.436)	0.0175	2.083 (1.043–4.157)	0.0375	2.320 (1.179–4.564)	0.0148
Malignancy (lung cancer vs. others)	5.121 (2.610–10.050)	<0.0001	4.885 (2.362–10.104)	<0.0001	5.807 (2.946–11.447)	<0.0001
Surgeon (surgeon A vs. others)	0.862 (0.273–2.718)	0.8000	0.872 (0.271–2.810)	0.8184		
Surgeon (surgeon B vs. others)	0.508 (0.171–1.511)	0.2234	0.86 (0.285–2.594)	0.7887

**Table 5:** Clinical characteristics of the patients with or without local infections

Variables	All patients	Local infection (−)	Local infection (+)	*P* value
*N*	34	23 (68 %)	11 (32 %)	
Gender—*n* (%)				0.4590
Female	12 (35 %)	7 (30 %)	5 (45 %)	
Male	22 (65 %)	16 (70 %)	6 (55 %)	
Age—mean ± SD	64.9 ± 11.1	66.9 ± 10.1	60.8 ± 12.3	0.1339
Age—*n* (%)				0.2714
Age ≤ 65 years	17 (50 %)	10 (43 %)	7 (64 %)	
Age > 65 years	17 (50 %)	13 (57 %)	4 (36 %)	
Malignancy (indication for the port)—*n* (%)				0.8519
Lung cancer	17 (50 %)	11 (48 %)	6 (55 %)	
Head and neck tumor	2 (6 %)	2 (9 %)	0 (0 %)	
Breast cancer	2 (6 %)	1 (4 %)	1 (9 %)	
Colorectal cancer	3 (9 %)	2 (9 %)	1 (9 %)	
Urological cancer	5 (15 %)	4 (17 %)	1 (9 %)	
Ovary cancer and cervical cancer	4 (12 %)	3 (13 %)	1 (9 %)	
Other malignancies	1 (3 %)	0 (0 %)	1 (9 %)	
Malignancy (lung cancer or others)—*n* (%)				0.7139
Lung cancer	17 (50 %)	11 (48 %)	6 (55 %)	
Other malignancies	17 (50 %)	12 (52 %)	5 (45 %)	
Surgeon—*n* (%)				0.6647
Surgeon A	11 (32 %)	7 (30 %)	4 (36 %)	
Surgeon B	19 (56 %)	14 (61 %)	5 (45 %)	
Other surgeons	4 (12 %)	2 (9 %)	2 (18 %)	
Pathogen—*n* (%)				
Gram-positive Bacteria	15 (44 %)	8 (35 %)	7 (64 %)	0.1512
*S. aureus*	13 (38 %)	6 (26 %)	7 (64 %)	0.0597
Gram-negative Bacteria	19 (56 %)	16 (70 %)	3 (27 %)	0.0301
*K. pneumoniae*	10 (29 %)	8 (35 %)	2 (18 %)	0.4375
*Candida spp*.	4 (12 %)	3 (13 %)	1 (9 %)	0.7379

## Discussion

The results of this study suggest that the type of venous port may affect the rate of BSIs. In addition, the patients who were older than 65 years or had lung cancer also had a significantly higher infection rate. Furthermore, we also found that the patients without signs of infection over the port had a significantly higher rate of gram-negative bacilli infections. The overall incidence of IVAP-related BSIs was 0.177 events/1000 catheter days, which is similar to previously reports (0.16 to 0.35 events/1000 port days) [2, 12, 14].

The Bard port is made of silicon, whereas the TYCO port is made of polyurethane. In our previous study, we found that the Bard fr 6.6 venous port had a significantly higher migration rate of up to 6.7 % compared with the Autosuture Chemosite fr 7.5 venous port (0 %) (*p* = 0.0006) because the Bard port was more flexible with a smaller caliber [15]*.* Silicone is a polymer that contains silicon, hydrogen, oxygen, and carbon, and silicone catheters are used for long-term vascular access (weeks to months), such as that required for the prolonged administration of chemotherapy. Polyurethane is a versatile polymer, and it is used in vascular catheters as it can provide enough tensile strength to allow for the catheter to pass through the skin and subcutaneous tissues without kinking. Polyurethane catheters are often used for short-term vascular cannulation, and they have been reported to have slightly higher colonization/infection rates although biofilm has been reported to form on both silicone and PU catheters [16].

The pathogenesis of IVAP-associated infections centers around multifaceted interactions between the bacteria, catheter, and host. Bacterial factors are probably the most important in the pathogenesis of infection, whereas catheter factors are the most modifiable with regard to preventing infection [17]. Bacterial adherence to catheters may depend, in part, on the nature of the biomaterial, and some substances eluted from the catheter may affect the viability and growth of different microorganisms [16, 18]. Catheters made of silicone have demonstrated a tendency towards an increased infection rate than those made of PU in some in vitro studies. However, these effects have only been demonstrated in vitro and in short-term studies using direct inoculation of bacteria onto the catheter [17, 19, 20]. To date, few studies have investigated late catheter-related infections with different catheter materials or infections following exposure to blood-borne bacteria in humans. Moreover, the time during which catheter materials have been shown to have an effect is very early, when catheter infection rates are already low [16, 19, 20]. Further large scale in vivo studies are needed to elucidate this issue.

In previous studies, the incidence of infection has been reported to be significantly higher in patients with hematologic malignancies, [8, 21, 22] with hematologic malignancies being more strongly related to delayed bloodstream infections than immediate local infections. The more intensive chemotherapy used for hematologic malignancies compared to that used for solid tumors may explain the increased infection rate [23]. However, no cases of IVAP-related BSIs in the patients with hematological malignancies were recorded in this retrospective study. Furthermore, the IVAP-related BSIs occurred mainly among the patients with lung cancer. Marín et al. indicated that in cases of bacteremia in patients with solid tumors, the most frequent neoplasms were hepatobiliary tumors (19 %), followed by lung cancer (18 %), and lower gastrointestinal malignancies (16 %) [9]. In addition, Anatoliotaki et al. reported that breast cancer (22 %) was most common followed by lung cancer (18 %) [11]. Lee et reported that the most common cancers in patients with IVAP-related BSIs were those of the lung, head, and neck [10].

Cutaneous microbial flora has been reported to play a major role in the IVAP-related BSIs that develop within a venous port [24], and gram-positive cocci are therefore considered to be the most prevalent organisms in patients with IVAP [3, 4, 25]. Gram-positive cocci may account for 50–80 % of IVAP-related BSIs [26]. In this study, *Staphylococcus aureus* was the most common single pathogen among all of the patients, and oxacillin-sensitive *Staphylococcu*s was more common than oxacillin-resistant *Staphylococcus*. However, half of the enrolled patients had gram-negative bacilli, the most common being *Klebsiella pneumoniae*, followed by *Acinetobacter baumannii*, *Escherichia coli*, and *Pseudomonas aeruginosa*. An increased incidence of central venous port-related bacteremia due to gram-negative pathogens has been reported in cancer patients [27, 28]. Furthermore, gram-negative bacilli, and especially glucose non-fermenting pathogens, tend to be the most common microorganisms accounting for port-related infections [7, 28]. Interestingly, Liaw et al. [13] reported that in patients with local inflammation, *Staphylococcus* species were the most common; however, in patients without local port inflammation, up to 91 % of the cases involved nosocomial glucose non-fermenting gram-negative bacilli, with *Acinetobacter baumannii* and *Enterobacter cloacae* being the most common. We further found that the patients with no signs of local venous port infection had a significantly higher rate of gram-negative bacilli. Based on these findings, empiric antibiotics for venous port infections should cover both gram-positive and gram-negative bacilli in patients with venous port infections but without signs of local infection.

There are several limitations to this study. First, it was a retrospective, single-hospital study, and the sample size was small. Second, some of the infections may have resulted from other sites of unrecognized infection. Third, not all patients had positive tip cultures in this study, and catheter tip cultures may not be sufficiently sensitive to diagnose port-related infections. Catheter tip cultures have been reported to be 100 % specific but less than 50 % sensitive for a diagnosis [29, 30].

## Conclusions

In conclusion, port type, age > 65 years, and lung cancer were all independent risk factors for IVAP-related BSIs. In addition, we also found that patients without signs of local infection over the port were significantly related to a higher rate of gram-negative bacilli infections.
